# Modifiable Arousal in Attention-Deficit/Hyperactivity Disorder and Its Etiological Association With Fluctuating Reaction Times

**DOI:** 10.1016/j.bpsc.2016.06.003

**Published:** 2016-11

**Authors:** Sarah-Naomi James, Celeste H.M. Cheung, Fruhling Rijsdijk, Philip Asherson, Jonna Kuntsi

**Affiliations:** a MRC Social, Genetic and Developmental Psychiatry Centre, Institute of Psychiatry, Psychology and Neuroscience, King’s College London, London; b Department of Psychology, Institute of Psychiatry, Psychology and Neuroscience, King’s College London, London; c Centre for Brain and Cognitive Development (CHMC), Department of Psychological Sciences, Birkbeck, University of London, London, United Kingdom

**Keywords:** ADHD, Arousal, Familial influences, Reaction time variability, Sibling study, Skin conductance

## Abstract

**Background:**

Cognitive theories of attention-deficit/hyperactivity disorder (ADHD) propose that high within-subject fluctuations of cognitive performance in ADHD, particularly reaction time (RT) variability (RTV), may reflect arousal dysregulation. However, direct evidence of arousal dysregulation and how it may account for fluctuating RTs in ADHD is limited. We used skin conductance (SC) as a measure of peripheral arousal and aimed to investigate its phenotypic and familial association with RTV in a large sample of ADHD and control sibling pairs.

**Methods:**

Adolescents and young adults (*N* = 292), consisting of 73 participants with ADHD and their 75 siblings, and 72 controls and their 72 siblings, completed the baseline (slow, unrewarded) and fast-incentive conditions of a RT task, while SC was simultaneously recorded.

**Results:**

A significant group-by-condition interaction emerged for SC level (SCL). Participants with ADHD had decreased SCL, compared with controls, in the baseline condition but not the fast-incentive condition. Baseline SCL was negatively associated with RTV, and multivariate model fitting demonstrated that the covariance of SCL with RTV, and of SCL with ADHD, was mostly explained by shared familial effects.

**Conclusions:**

ADHD is associated with decreased, but modifiable, tonic peripheral arousal. A shared familial cause underlies the relationship between arousal and RTV and between arousal and ADHD. Given the malleability of SCL, if our findings are replicated, it warrants further exploration as a potential treatment target for ADHD.

Attention-deficit/hyperactivity disorder (ADHD) has long been proposed to link to problems with the arousal system. Cognitive theories of ADHD, such as the state regulation model (1, 2) or more recent dual-process models (3, 4, 5), propose that the high within-subject fluctuations of cognitive performance in ADHD may reflect problems in regulating arousal. Yet, direct objective evidence of arousal dysregulation and how it may account for fluctuating cognitive performance in ADHD is limited to date.

Measuring skin conductance (SC) provides an objective, reliable measurement of arousal in the peripheral nervous system (6). SC sensitively measures electrical changes in electrodermal activity, which is stimulated by the autonomic sympathetic nervous system, a key system in influencing arousal and alertness (6, 7, 8). Two commonly used measurements of SC are skin conductance level (SCL), which represents a tonic level of arousal (averaged over a given time window), and skin conductance response (SCR) amplitude, which represents a phasic (transient) event-related change in SC (9). Increased SCL indexes an increase in peripheral arousal, whereas increased SCR amplitude indicates a stronger, higher intensity arousal response (6). Although early studies of SC in ADHD yielded conflicting findings (10, 11, 12, 13), a number of more recent studies, benefiting from advancements in SC technique, report attenuated SCL in children with ADHD at rest and in task conditions, indicating hypoarousal (14, 15, 16, 17, 18, 19, 20, 21). However, discrepancies still remain because some studies report no differences in SCL between adults with and without ADHD (22, 23).

The aspect of cognitive performance that most strongly fluctuates in people with ADHD is their speed of responding on standard reaction time (RT) tasks, leading to high RT variability (RTV) (24, 25, 26). Our previous analyses on a large sample of ADHD and control sibling pairs showed how RTV captured a large proportion of the familial influences underlying ADHD and separated from a second familial cognitive impairment factor that captured executive function impairments, such as response inhibition (27). In twin analyses the genetic association of RTV was observed particularly strongly with inattention symptoms (28). RTV can, however, improve in individuals with ADHD under certain circumstances: a meta-analysis of eight studies of varying designs suggested an overall significant, although small, effect of incentives (24). While most of these studies have rewarded successful inhibition, we have examined the effects of rewarding specifically on a reduction in RTV and have further combined the effects of rewards with a faster event rate, to maximize potential RTV improvement. Under such conditions, using the Fast task, we have consistently observed ADHD-sensitive improvement in RTV from baseline to a fast-incentive condition (25, 29, 30).

Applying SC measurement in a study on ADHD, O’Connell *et al.* (31) investigated performance on a sustained attention to response task. SC was measured before and after taking part in either self-alert training, whereby participants learned to modulate their own arousal levels, transiently increasing their arousal at regular intervals with the aim of reducing momentary lapses of attention, or a placebo training condition. Compared with pretraining performance, ADHD and control adult participants with the alertness training had increased SCR, indicating increased transient arousal; had a more consistent RTV over testing sessions; and made fewer commission errors. Contrarily, ADHD participants and controls in the placebo training condition, who were not taught to modulate their arousal levels, had decreased SCR with time, indicating a decrease in stimulus-related arousal, as well as increased RTV, compared with their pretraining performance. Although the investigators did not report correlations between SC and the cognitive performance measures, they note that SC and RTV followed a similar pattern: block-by-block increases in RTV were accompanied by gradual decreases in SCR, indicating a drop in arousal response over time (31).

We aimed to perform a detailed investigation of SC as an objective measure of peripheral arousal, and its potential association with fluctuating RTs in a large sample of ADHD and control sibling pairs. First, we aimed to investigate if people with ADHD differ from controls in SCL and SCR amplitude during baseline (slow, unrewarded) RT performance. Second, we aimed to test if a fast-incentive condition increases SC-indexed arousal, and if it does, whether it increases more in the ADHD group than in the control group. Third, for the SC variables that show group differences, we aimed to investigate their familial association with RTV and ADHD diagnosis, using sibling model fitting analyses, and to consider specific causal models that may explain the relationships that emerge.

## Methods and Materials

### Sample

Participants are members of the Sibling EEG Follow-Up Study (SEFOS) (32, 33, 34), which investigates neurophysiological and cognitive measures in a follow-up sample of ADHD and control sibling pairs. ADHD and control participants who had taken part in our previous research (27, 35) were invited to take part in this study. ADHD participants were included if they had ADHD in childhood and met DSM-IV criteria for any ADHD subtype at follow-up. Exclusion criteria included IQ < 70, autism, epilepsy, brain disorders, and any genetic or medical disorder associated with externalizing behaviors that might mimic ADHD. The investigation was performed in accordance with the latest version of the Declaration of Helsinki.

From the original follow-up sample of 404 participants, 311 had SC measured (because SC data collection only started after initial participants had already been assessed). We excluded from the analyses 10 ADHD participants [SC equipment failure (*n* = 9), extreme drowsiness (*n* = 1)] and 9 control participants [SC equipment failure (*n* = 8) and met ADHD criteria based on parent report (*n* = 1)]. The final sample consisted of 73 ADHD probands [mean (SD) age, 18.3 (2.9) years; 87% male], 75 siblings of ADHD probands [mean age, 18.3 (2.9) years; 48% male], 72 controls [mean age, 17.48 (1.8) years; 94% male], and 72 control siblings [mean age, 17.11 (2.4) years; 68% male].

For the ADHD control group differences analyses (aims 1 and 2), both members of control sibling pairs formed the control group (*n* = 144); siblings of ADHD probands were excluded unless they had an ADHD diagnosis themselves. For these analyses, the ADHD and control groups did not differ in sex (χ^2^ = 1.64, *p* < .20) but did differ in age (*t* = 0.54, *p* = .04) and IQ (*t =* 6.01, *p* < .001). In all these analyses we included age as a covariate, and in additional analyses we added IQ as a second covariate. For the model fitting analyses (aim 3), all participants were included and differed in age (*t* = 1.97, *p* = .05), sex (χ^2^ = 35.2, *p* < .01), and IQ (*t =* 22.46, *p* < .01). In these analyses we therefore used age and sex as covariates, with additional analyses also including IQ as a further covariate. All participants were of European Caucasian descent.

### Procedure

The Fast task was administered as part of a longer assessment session at the research center. For participants prescribed stimulants, a 48-hour ADHD medication-free period was required. Participants abstained from caffeine, smoking, and alcohol on the day of testing. Face-to-face or telephone clinical interviews were administered to the parent of each ADHD proband shortly before or after the participant’s assessment.

### Measures

#### IQ

The vocabulary and block design subtests of the Wechsler Abbreviated Scale of Intelligence (36) were administered to all participants to derive an estimate of IQ.

#### ADHD Diagnosis

The Diagnostic Interview for ADHD in Adults (DIVA) (37), a semistructured interview based on the DSM-IV criteria, was conducted with the parent for current symptoms only, because in all cases a clinical and research diagnosis of combined type ADHD had already been established (35). The Barkley’s Functional Impairment Scale (38) was used to assess functional impairments commonly associated with ADHD in five areas of their everyday life. Each item ranges from 0 (never or rarely) to 3 (very often). Participants were classified as affected, if they scored a yes on six or more items on the Diagnostic Interview for ADHD in Adults for either inattention or hyperactivity-impulsivity based on parent report, and scored ≥2 on two or more areas of impairments on the Barkley’s Functional Impairment Scale, rated by their parent.

### The Fast Task

The slow-unrewarded (baseline) condition consists of 72 trials, which followed a standard warned four-choice RT task. Four empty circles (warning signals, arranged horizontally) first appeared for 8 seconds, after which time one of them (the target) was colored in. Participants were asked to press the response key that directly corresponded to the position of the target stimulus. After a response, the stimuli disappeared from the screen, and a fixed intertrial interval of 2.5 seconds followed. Speed and accuracy were emphasized equally in the task instructions. If the participant did not respond within 10 seconds, the trial terminated. A comparison condition of 80 trials with a fast event rate (fore-period of 1 second) and incentives followed the baseline condition (29). The fast-incentive condition is always administered after the baseline condition. SC measures and cognitive performance measure (RTV) from each condition was included in this analysis. Owing to the longer fore-period in the slow condition, the two conditions were not matched on task length, but they were matched on the number of trials. We analyzed RTV and SC performance both on the full slow condition and between three 4-minute length-matched segments (Supplemental Tables S1 and S2) (29).

#### Skin Conductance

SC data were measured by attaching a pair of reusable 8-mm-diameter silver-silver chloride electrodes on the palm of the hand (thenar eminence and hypothenar eminence) of the participant’s nondominant hand at the start of the testing session. A nonsaline gel was used to increase impedance and to help establish an electrical signal. A constant imperceptible voltage (0.5 V) was applied.

SC was recorded using PSYCHLAB SC5 24-bit equipment system, which has an absolute accuracy of ±0.1 microsiemen (PSYCHLAB, London, UK). The SC5 was connected to a computer to run the PSYCHLAB software, where were monitored, recorded in real time, and automatically digitized. Stimulus onset and participant response events were recorded on a common timeline, which enabled SC activity to be stimulus locked.

SC data values were calculated using a SC system that is based on a SC sigmoid-exponential model that allows the tonic measure of SCL to be disentangled from phasic, stimulus-associated SCRs and further allows the decomposition of overlapping SCRs (6, 9, 39, 40). This system, therefore, is appropriate to use in conditions with long and short interstimulus intervals (41). The statistical model was applied to each condition, as a whole. SCR amplitude (change in SC from the baseline to the highest point of the SCR) was derived from this method and was calculated on a trial-by-trial basis. The criteria for the smallest SCR were set at 0.02 microsiemen. Means of SC variables (SCL and SCR amplitude) were calculated per participant, across each condition.

### Analyses

#### Covariates

Age was used as a covariate in all analyses. Analyses were initially performed without controlling for IQ, but we subsequently reran all analyses with IQ as a covariate to examine IQ effects. Sex was not included as a covariate in the group analyses to avoid controlling for ADHD status (32). Instead, we explored the effect of sex by rerunning all analyses with the female participants (*n* = 15) removed; the pattern of results remained the same (results are available on request). Analyses were rerun using anxiety and depression scores from the Clinical Interview Schedule-Revised (42) as additional covariates to investigate their confounding effects, but the significance of the results did not change (Supplemental Table S6). All variables were skewed and transformed using the optimized minimal skew (lnskew0) command in Stata version 11.1 (Stata Corp., College Station, TX). Tests assessing sphericity and equality of variances were performed to ensure that assumptions were met.

#### ADHD-Control Group Comparisons

To test for main effects of group (ADHD vs. controls), condition (baseline vs. fast-incentive), and interactions for SC variables and RTV, the data were analyzed using random intercept models and logistic regression in Stata. The random intercept model is a multilevel regression model that can be used as an alternative to analysis of covariance to control for genetic relatedness (where both siblings from a pair are included in analyses) in a repeated-measures design, using a robust cluster command to estimate standard errors (32, 43, 44).

#### Structural Equation Modeling on Sibling Data

Structural equation modeling in OpenMx (45) was used on sibling-pair data to decompose the variance of traits into etiological factors. Whereas in twin studies comparison between monozygotic and dizygotic twin pairs enables estimation of additive genetic, shared environmental, and nonshared environmental influences, sibling pairs (all sharing 50% of their alleles and 100% of the environment they grow up in) only enable estimation of the combined effects of additive genetic and shared environmental influences (familial [F] effects). In addition to F effects, nonshared effects (NE) are estimated, representing effects due to nonshared environment/genes as well as possible measurement error.

Multivariate modeling on sibling data uses the additional cross-sib cross-trait information to decompose the observed phenotypic correlation (Rph) between traits into etiological factors. Similar sibling design analyses have been previously performed by our group [see (46) for a more detailed description and rationale of the analysis]. In addition, by using the correlations between the F and NE factors, and the standardized estimates, we calculated the extent to which the Rph between any two variables is due to F (Rph-F) and NE (Rph-NE).

#### Phenotypic Correlations

Before FNE modeling (described in the section above), sibling correlations were estimated from a constrained correlation model to give maximum likelihood estimates of correlations between the traits within and across pairs while applying some constraints. Applied constraints reflect the assumptions of the familial model, that is, that phenotypic correlations across traits within individuals are the same across siblings and that cross-trait cross-sibling correlations are independent of sibling order. Variables used in the sibling model fitting were selected by running phenotypic correlations on variables that showed group differences, and only variables that had a significant relationship with RTV were further analyzed.

#### Phenotypic Mediation Model

To further investigate a more etiological model that may account for the relationship between SC variables that are associated with both RTV and ADHD, and given the theoretical scope that RTV (an observed behavioral response) may reflect hypoarousal (an internal physiological process), we hypothesized that RTV may mediate the relationship between SC-indexed arousal and ADHD. A phenotypic mediation model was fitted with SC variables that may be causally associated with both RTV and ADHD. Significant (partial) mediation occurs when a third variable explains some of the association between two other variables (47). The phenotypic mediation model was specified to account for the sibling-structure and selected nature of the data using similar constraints as the correlation model described above. The phenotypic relationship across traits within individuals is specified by means of causal paths, which were constrained to be equal across siblings. The sibling-structure was accounted for by specifying correlational paths across sibling variables.

#### Ascertainment Correction

To account for the selected nature of the sample (selection on ADHD probands), the selection variable (ADHD status) was included in all models with its variables fixed to population-known values. In the correlation and mediation model this involves fixing the sibling correlation for ADHD status to 0.40 and in the FNE models fixing *F* to 0.40, representing 80% genetic variance (in case shared environment = 0). In addition, the threshold on ADHD liability was fixed to a *z* value of 1.64 to correspond to a population prevalence of 5% [see Rijsdijk *et al.* (48) for further explanation and validation of this approach].

## Results

### ADHD-Control Group Comparisons

**Figure 1:**
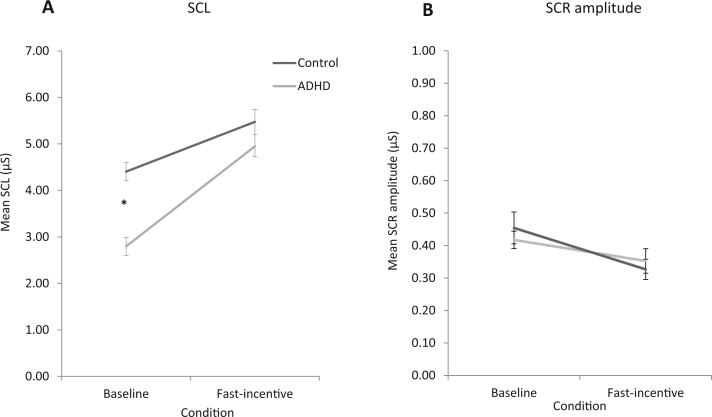
Skin conductance variables [**(A)** skin conductance level (SCL) and **(B)** skin conductance response (SCR) amplitude] measured in control (black) and attention-deficit/hyperactivity disorder (ADHD; gray) groups during performance on the baseline and fast-incentive conditions of the Fast task. *p < .05 significance.

For SCL data, a random intercept model indicated a significant main effect of condition (*z* = 8.95, *p* = .01) and group-by-condition interaction (*z* = 1.89, *p* = .04), but no main effect of group (*z* = .19, *p* = .85) (Figure 1A). Post hoc regression analyses revealed that compared with controls, individuals with ADHD showed significantly lower SCL in in the baseline condition (*t* = −5.64, *p* < .001), but not in the fast-incentive condition (*t* = 1.10, *p* = .27) (Table 1). Both ADHD and control groups had a significant within-group increase from the baseline to fast-incentive condition (*t* = 7.52, *p* < .01; *t* = 6.44, *p* < .01, respectively), but the ADHD group had a greater increase than controls (*t* = 1.94, *p* < .05).

For SCR amplitude data, a random intercept model showed no significant main effects of group (*z* = .46, *p* = .61), condition (*z* = .42, *p* = .28) or group-by-condition interaction (*z* = .69, *p* = .51) (Figure 1B).

All group analyses were rerun with IQ as a covariate, but the significance of results remained unchanged. Analyses were rerun using three length-matched segments from the baseline condition and testing them separately against the fast-incentive condition, but the significance of results did not change (Supplemental Tables S1 and S2). Although our sample had a 48-hour medication-free period, to explore the longer-term use of medication, we ran the following additional analyses: 1) SC comparison tests between unmedicated and medicated participants with ADHD, 2) using current stimulant medication as an additional covariate, and 3) analyses in unmedicated participants only. The significance of results did not change in any additional analyses (Supplemental Tables S3, S4, and S5).

We ran additional phenotypic correlations to examine the SCL-RTV and SCR-RTV relationship in ADHD and control groups separately (Supplemental Table S7). In the baseline condition, lower SCL significantly predicted higher RTV in the ADHD group (*r* = −.31, *p* < .01), but this correlation did not reach significance in the control group (*r* = −.12, *p* = .15), and Fisher’s *z* test indicated that the correlations between the groups differed from one another at a trend level (*z* = −1.37, *p* = .08). In the fast-incentive condition, the RTV-SCL correlations were not significantly different between the groups (*z* = −.97, *p* = .16; *r* = −.29, *p* < .01 in the ADHD group; *r* = −.16, *p* = .06 in the control group). There were no significant SCR-RTV correlations.

### Familial Association Between SCL, RTV, and ADHD

Given that SCL showed a significant group-by-condition interaction, a significant correlation with RTV with large effect sizes and the biggest significant group difference in the baseline condition, we next investigated the phenotypic and etiological overlap between SCL, RTV, and ADHD in the baseline condition. The maximum likelihood phenotypic, cross-sibling, and cross-sibling-cross-trait correlations across SCL, RTV, and ADHD are presented in Table 2.

**Figure 2:**
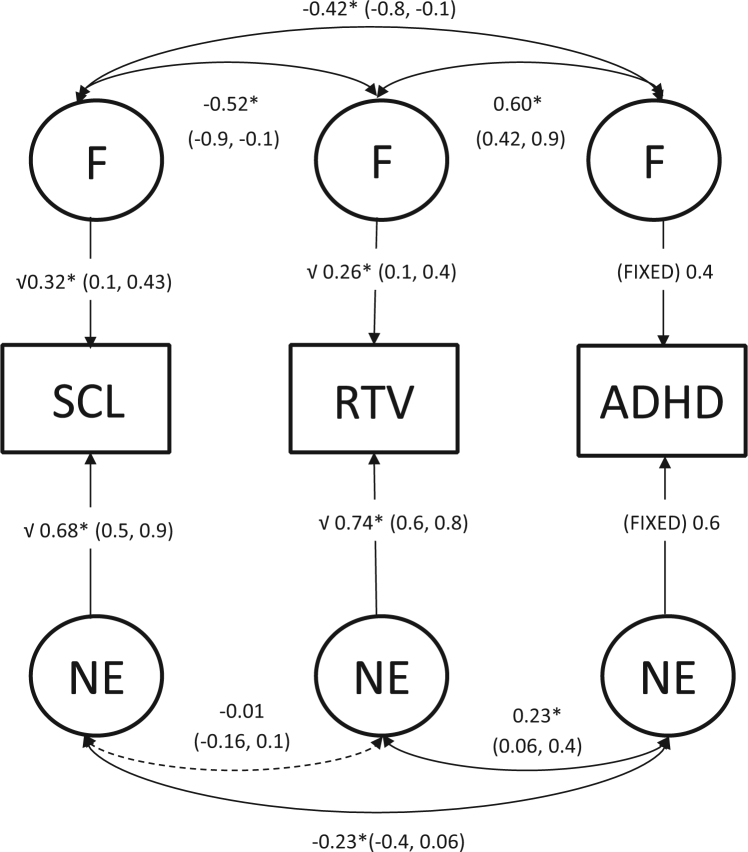
Standardized solution of the full correlated factor model across skin conductance level (SCL), reaction time variability (RTV), and attention-deficit/hyperactivity disorder (ADHD) in the baseline condition. Solid lines and asterisks depict significant paths (*p* ≤ .05) and dotted lines depict nonsignificant paths (*p* > .05). Confidence intervals are indicated in parentheses. F, familial effects; NE, nonshared effects.

Sibling-pair multivariate model fitting was performed to decompose variance/covariance of traits into etiological factors F and NE (Figure 2). We calculated the extent to which the phenotypic correlation between any two variables is due to F (Rph-F) and NE (Rph-NE) and express these contributions as a percentage (Table 3). Shared familial influences accounted for 55% of the total phenotypic correlation between SCL and ADHD, 94% of the phenotypic correlation between SCL and RTV, and 59% of the phenotypic correlation between ADHD and RTV.

### Phenotypic Mediation Model

**Figure 3:**
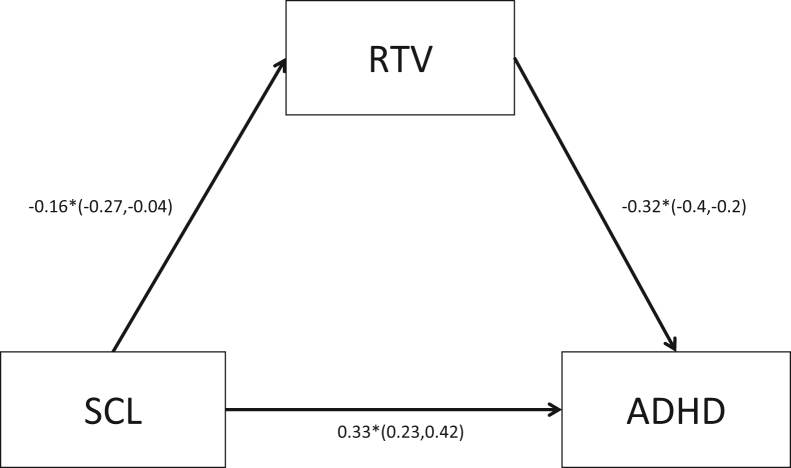
Reaction time variability (RTV) as a mediator of skin conductance level (SCL) and attention-deficit/hyperactivity disorder (ADHD) in the baseline condition. Solid lines and asterisks depict significant paths (*p* ≤ .05), and dotted lines depict nonsignificant paths (*p* > .05). Confidence intervals are indicated in parentheses.

Given the significant phenotypic and familial relationship of baseline SCL with RTV and with ADHD, we tested whether baseline RTV mediated the relationship between baseline SCL and ADHD status. In the mediation model, the causal paths specified were all significant, and partial mediation by RTV was indicated (Figure 3). However, model fit statistics demonstrate that the causal mediation model was not a good fit (Bayesian information criterion = 2511, root mean square error of approximation = 0), which is demonstrated by a significant χ^2^ statistic (−Δχ^2^ *=* −70.09, Δdf = 1, *p* < .01).

## Discussion

In a large sibling study of 292 participants, we show that tonic peripheral arousal, indexed with SCL, is decreased in young people with ADHD during performance on a baseline RT task but normalizes in a faster condition with incentives, indicating modifiable arousal dysregulation in ADHD. We further show that a substantial degree of familial sharing accounts for the significant phenotypic associations between SCL and RTV and between SCL and ADHD.

The SC measure associated with ADHD was SCL. Lower SCL during baseline RT performance indicated a lower tonic level of peripheral arousal in individuals with ADHD, consistent with accounts of hypoarousal (14, 15, 16, 21, 49). No group differences emerged for SCR amplitude. Although SCL and SCR are commonly used measurements of SC, they are thought to index different processes (50). For example, neuroimaging studies show that the activity of the ventromedial prefrontal cortex and orbitofrontal cortex is associated with SCL (51), whereas anterior prefrontal cortex and limbic regions are associated with SCR (50, 51). Our results, therefore, suggest that although the processes involved in tonic level of peripheral arousal (SCL) are impaired in individuals with ADHD during baseline performance, the processes involved in the phasic, discrete, arousal response elicited by stimulus onset (SCR amplitude) are not affected. The separation that we observed between SCL and SCR amplitude in their association with ADHD is also supported by studies suggesting that treatment with methylphenidate, an effective medication used to reduce ADHD symptoms, is associated more directly with increased SCL arousal (12, 21, 52).

Tonic peripheral arousal (SCL) normalized in the ADHD group in the fast-incentive condition, as indicated by a significant group-by-condition interaction and lack of a group difference in the fast-incentive condition. The malleability of SCL is in line with results of modifiable SC-indexed arousal (16, 21, 31) and resembles the pattern observed for RTV (30). The overall pattern of findings is therefore suggestive of an arousal dysregulation rather than stable hypoarousal, in individuals with ADHD.

To investigate the familial association between SCL and RTV directly, we focused on the baseline condition that is most sensitive to ADHD. The SCL-RTV correlation was largely (94%) accounted for by shared familial influences, demonstrating that the association of underarousal with attentional fluctuations is mostly due to overlapping familial effects. Of the familial influences on RTV, half were correlated with those on SCL, indicating that peripheral arousal captures half of the familial influences that contribute to the attentional fluctuations. These findings are in line with theories linking RTV to arousal dysregulation (4, 29, 53, 54, 55, 56). However, because half of the familial influences on RTV were not correlated with those on SCL, this implies there are also nonoverlapping, distinct, familial influences that contribute to RTV, in line with a multifactorial etiology of increased RTV (53).

We further investigated the familial association between SCL and ADHD and found that shared familial effects accounted for 59% of the phenotypic correlation between them, providing further support for an etiological link between underarousal and ADHD. Of the familial influences on ADHD, a third correlated with those on SCL, demonstrating that peripheral arousal captures a third of the familial influence contributing to ADHD. However, two thirds of the familial influences on ADHD did not correlate with those on SCL, implying that there are also nonoverlapping familial influences that contribute separately to ADHD. These findings are in agreement with the view that arousal dysregulation is not the only contributing factor to ADHD, in line with the multifactorial nature of ADHD (3, 4, 5, 55, 56, 57).

In a novel attempt to investigate the causal pathways that underlie the phenotypic relationship between SCL-indexed arousal, RTV, and ADHD, we fitted a model that tests whether there are causal pathways from 1) SCL to RTV and 2) RTV to ADHD and 3) whether RTV mediates the association between SCL-indexed arousal and ADHD, or whether there is a direct causal pathway from SCL to ADHD. The mediation and causal paths between all variables were significant, suggesting that there are two pathways from SCL-indexed arousal to ADHD: an indirect causal pathway from arousal to RT fluctuations to ADHD and a direct casual pathway from arousal to ADHD. Overall, our statistical model is consistent with ADHD theories that suggest a role for arousal dysregulation in the etiology of ADHD and the observed lapses of attention (3, 4, 5). It is further suggestive of complex relationships between the variables; although the association between underarousal and ADHD was partially mediated by attentional fluctuations (RTV), underarousal had additional direct influences on ADHD. However, the causal mediation model did not fit the data well; therefore, these causal pathway results should be interpreted with caution and further explored in future research.

Because this is the first family study on skin conductance and ADHD to our knowledge, our findings await replication. SC should also be studied in relation to other cognitive tasks, to investigate the generalizability of the findings. In addition, twin studies are required to establish whether the familial influences we identified reflect largely shared genetic rather than shared environmental influences; because previous research suggests a limited role for shared environmental effects for ADHD (58), SC (59, 60), and RTV (61), a strong genetic component seems likely.

In conclusion, we identify SCL as an informative index of underlying, malleable hypoarousal in ADHD. The demonstration of a link between SCL, RTV, and ADHD provides physiological support for the arousal dysregulation accounts (1, 2, 3, 4, 5). If our findings are replicated in future research, SCL warrants further exploration as a potential treatment target.

## Figures and Tables

**Table 1:** Descriptive Statistics of Sex, IQ, Age, RTV, and SC Measures and Group Comparisons Between the Control and ADHD Group

	Control	ADHD Probands	Group Comparisons		Effect Size of Group Comparison	
			*t/F*	*p*	Cohen’s *d*	Cohen’s *d:* IQ Controlled
Demographic Characteristics						
Male sex, %	81	87	1.64	.20		
IQ, Mean (SD)	109.60 (12.52)	98.60 (14.50)	601.00	<.01		
Age, Years, Mean (SD)	17.30 (2.15)	18.30 (2.90)	0.54	.60		
RTV, Mean (SD)						
Baseline	3.80 (0.40)	4.70 (0.80)	6.59	.001	−1.20	−0.95
Fast-Incentive	3.33 (0.60)	3.70 (0.70)	1.49	.14	−0.90	−0.90
SCL, Mean (SD)						
Baseline	1.84 (0.30)	1.56 (0.30)	−5.64	.001	0.72	0.67
Fast-Incentive	3.20 (2.00)	3.70 (2.10)	1.10	.27	−0.17	−0.15
SCR Amplitude, Mean (SD)						
Baseline	0.41 (0.30)	0.45 (0.60)	1.32	.20	−0.06	−0.06
Fast-Incentive	0.34 (0.20)	0.32 (0.23)	0.07	.91	0.05	0.03

Age has been controlled for in the analyses on SC and RT variables. Cohen’s effect sizes (*d*) are presented without and with IQ as a covariate. Group means of transformed data and subsequent group comparison tests are listed.

ADHD, attention-deficit/hyperactivity disorder; RT, reaction time; RTV, reaction time variability; SC, skin conductance; SCL, skin conductance level; SCR, skin conductance response.

**Table 2:** Maximum-Likelihood Phenotypic, Cross-Sibling and Cross-Sibling Cross-Trait Correlations Across Baseline SCL, RTV, and ADHD

Correlations	*R*	95% CI
Phenotypic Correlations Within Individual		
SCL-RTV	−.15^a^	(−0.23, −0.01)
SCL-ADHD	−.31^a^	(−0.42, −0.19)
RTV-ADHD	.35^a^	(0.23, 0.46)
Cross-Sibling Correlations		
SCL	.26^a^	(0.07, 0.40)
RTV	.26^a^	(0.10, 0.40)
ADHD	Fixed .40	
Cross-Sibling-Cross-Trait Correlations		
SCL-RTV	−.15^a^	(−0.24, −0.01)
SCL-ADHD	−.14^a^	(−0.27, −0.02)
RTV-ADHD	.20^a^	(0.07, 0.30)

ADHD, attention-deficit/hyperactivity disorder; CI, confidence interval; RTV, reaction time variability; SCL, skin conductance level.

a *p* < .05.

**Table 3:** Phenotypic Correlations (Rph) and the Phenotypic Correlations due to Familial Effects (Rph-F) and Nonshared Effects (Rph-NE) Across SCL, RTV, and ADHD

	Rph (95% CI)	Rph-F (% Contribution)	Rph-NE (% Contribution)
SCL-RTV	−0.15^a^ (−0.25, −0.02)	−0.14 (94)	−0.01 (6)
SCL-ADHD	−0.31^a^ (−0.39, −0.16)	−0.17 (59)	−0.14 (41)
RTV-ADHD	0.35^a^ (0.23, 0.45)	0.20 (57)	0.15 (43)

ADHD, attention-deficit/hyperactivity disorder; CI, confidence interval; RTV, reaction time variability; SCL, skin conductance level.

a *p* < .05.
