# Supplementary material for: Modifiable Arousal in Attention-Deficit/Hyperactivity Disorder and Its Etiological Association With Fluctuating Reaction Times
Source: Biol Psychiatry Cogn Neurosci Neuroimaging. 2016 Nov;1(6):539–47. doi: 10.1016/j.bpsc.2016.06.003 (PMC5094448; doi:10.1016/j.bpsc.2016.06.003)
Supplement: Supplementary file 1 — Supplementary material [file mmc1.pdf]

Modifiable Arousal in Attention-Deficit/Hyperactivity Disorder and Its Etiological Association With Fluctuating Reaction Times

Supplemental Information

Table S1. Means and group differences in SCL and SCR amplitude between 3 four-minute segments of baseline condition and the fast-incentive condition (controls = 144, ADHD = 73). Baseline 1: 0-3.59 mins, Baseline 2: 4-7.59 mins, Baseline 3: 8-11.59 mins. Fast–incentive condition: 0-4 minutes.

	**Control**	**ADHD probands**	**Group comparisons**	
	Mean	Mean	*t*	*p*
SCL				
Baseline 1	1.86	1.56	2.41	0.01*
Baseline 2	1.83	1.52	1.99	0.03*
Baseline 3	1.79	1.51	2.10	0.03*
Fast-incentive	3.20	3.70	1.10	0.27
SCR amplitude				
Baseline 1	0.41	0.40	0.25	0.40
Baseline 2	0.37	0.37	1.21	0.20
Baseline 3	0.39	0.41	0.52	0.60
Fast-incentive	0.34	0.32	0.07	0.91

Skin conductance level (SCL), skin conductance response (SCR) amplitude. Group means of transformed data and subsequent group comparison tests are listed. *$p < 0.05$.

Table S2. Main effect of group (ADHD vs control), condition (segment of baseline condition vs the fast-incentive condition), and group x condition interaction, controlling for age (controls = 144, ADHD = 73). Baseline 1: 0-3.59 mins, Baseline 2: 4-7.59 mins, Baseline 3: 8-11.59 mins. Fast–incentive condition: 0-4 minutes.

**Condition**	**Main effects**	***t***	***p***
SCL			
Baseline 1 vs fast-incentive	group	1.37	0.17
	condition	4.58	0.01
	group x condition	2.48	0.01
Baseline 2 vs fast-incentive	group	1.22	0.22
	condition	4.59	0.01
	group x condition	2.21	0.04
Baseline 3 vs fast-incentive	group	1.55	0.12
	condition	2.10	0.03
	group x condition	2.03	0.04
SCR amplitude			
Baseline 1 vs fast-incentive	group	1.39	0.16
	condition	0.81	0.42
	group x condition	0.75	0.45
Baseline 2 vs fast-incentive	group	0.95	0.34
	condition	0.52	0.61
	group x condition	0.45	0.34
Baseline 3 vs fast-incentive	group	0.78	0.30
	condition	0.55	0.56
	group x condition	0.69	0.49

Skin conductance level (SCL), skin conductance response (SCR) amplitude.

Table S3. Means and group differences in SCL and SCR amplitude between unmedicated and medicated ADHD participants in the baseline condition and the fast-incentive condition (unmedicated = 35, medicated = 38), controlling for age.

	**Unmedicated ADHD**	**Medicated ADHD**	**Group comparisons**	
	Mean (SD)	Mean (SD)	*t*	*p*
SCL				
Baseline	1.66 (0.40)	1.56 (0.38)	0.67	0.50
Fast-incentive	5.27 (1.54)	5.49 (2.01)	0.76	0.45
SCR				
Baseline	-0.94 (0.49)	-0.83 (0.59)	-0.68	0.49
Fast-incentive	-1.32 (0.65)	-1.26 (0.76)	0.06	0.95

Note: raw scores are reported. Skin conductance level (SCL), skin conductance response (SCR) amplitude.

Table S4. Main effect of group (ADHD vs control), condition (whole baseline condition vs fast-incentive condition), and group x condition interactions, controlling for age and stimulant medication use (controls = 144, ADHD = 73).

Main effect	*t*	*p*
SCL		
Group	0.10	0.91
Condition	29.94	<0.01
Group x condition	2.55	0.01
SCR amplitude		
Group	0.21	0.84
Condition	0.37	0.71
Group x condition	0.71	0.48

Skin conductance level (SCL), skin conductance response (SCR) amplitude.

Table S5. Main effect of group (ADHD vs control), condition (whole baseline condition vs fast-incentive condition), and group x condition interactions in an un-medicated sample (controls = 144, ADHD = 35), controlling for age.

**Main effect**	***t***	***p***
SCL		
Group	1.47	0.14
Condition	20.88	<0.01
Group x condition	2.24	0.01
SCR amplitude		
Group	0.07	0.94
Condition	0.25	0.80
Group x condition	0.98	0.33

Skin conductance level (SCL), skin conductance response (SCR) amplitude.

Table S6. Main effect of group (ADHD vs control), condition (whole baseline condition vs fast-incentive condition), and group x condition interactions controlling for age, and anxiety, and depression scores (controls = 144, ADHD = 73). Anxiety and depression scores are taken from the Clinical Interview Schedule-Revised (CIS-R).

**Covariates**	**ADHD mean (SD)**	**Control Mean (SD)**	***t***	***p***
Anxiety	*0.45 (0.93)*	*0.19 (0.54)*	2.00	0.05
Depression	*0.31 (0.80)*	*0.22 (0.56)*	0.73	0.47

**Covariates**	**Main effect**	***t***	***p***
Age and anxiety score			
	SCL		
	Group	0.58	0.56
	Condition	23.63	<0.01
	Group x condition	2.47	0.01
	SCR amplitude		
	Group	1.35	0.14
	Condition	0.87	0.49
	Group x condition	0.78	0.48
Age and depression score			
	SCL		
	Group	0.58	0.56
	Condition	23.63	<0.01
	Group x condition	2.47	0.01
	SCR amplitude		
	Group	1.31	0.19
	Condition	0.88	0.41
	Group x condition	0.72	0.47

Skin conductance level (SCL), skin conductance response (SCR) amplitude.

Table S7. Pearson correlations in ADHD and control groups separately, between skin conductance level (SCL), skin conductance response (SCR) amplitude and reaction time variability (RTV), in the baseline and fast-incentive condition.

	**SCL**	**SCR amplitude**
Control		
RTV-baseline	-0.12	-0.10
RTV-fast-incentive	-0.16	-0.02
ADHD		
RTV-baseline	-0.31**	-0.09
RTV-fast-incentive	-0.29**	-0.32

** $p < 0.01$ skin conductance level (SCL), skin conductance response (SCR) amplitude.
